# Improving syndromic testing in oncology: performance characteristics of multiplex PCR assays for evaluation of infectious gastroenteritis among hospitalized cancer patients

**DOI:** 10.1017/ash.2026.10776

**Published:** 2026-07-07

**Authors:** Justin Laracy, Judy Yan, Rich Kodama, Shauna Usiak, Emily Walits, Tania Bubb, Mini Kamboj

**Affiliations:** Memorial Sloan Kettering Cancer Center, USA

## Abstract

**Background:**

Multiplex gastrointestinal polymerase chain reaction (GI-PCR) tests of the stool for the evaluation of infectious diarrhea are prone to false-positive target detections. However, there is no recommended testing approach in immunocompromised cancer patients who are often excluded from diagnostic stewardship protocols. The aim of this study was to evaluate the performance characteristics of the BioFire® FilmArray® GI-PCR and assess whether the findings support a diagnostic stewardship approach to limit testing among hospitalized cancer patients.

**Methods:**

A retrospective study of 29,727 GI-PCR tests was performed at Memorial Sloan Kettering Cancer Center in New York City, evaluating data from February 1, 2020, to January 29, 2025. All tests sent from all locations and across all patient types were included in the study.

**Results:**

The overall GI-PCR positivity rate was greatest in the outpatient setting at 23.4% and dropped at 15.6% in the early stage of admission (hospital days 0–2) and 10.1% in the late stage of admission (hospital day 3 and beyond). Across all subgroups analyzed, norovirus and Enteropathogenic *E. coli* (EPEC) were the most frequently detected targets with all remaining targets detected at rates below 2%. The low pathogen detection rate was preserved among patients with neutropenia and recipients of recent cellular therapy.

**Conclusion:**

BioFire FilmArray GI panel has low utility in the inpatient setting including for severely immunosuppressed hosts. These observations identify a potential opportunity for diagnostic stewardship and suggest that commonly applied testing restrictions may warrant further evaluation in immunocompromised oncology populations.

## Introduction

The advent of rapid and accessible molecular assays has transformed the field of infectious diseases diagnostics in the form of bundled syndromic testing. Compared to conventional testing, syndromic panels such as multiplexed polymerase chain reaction (PCR) offer unparalleled diagnostic efficiency by consolidating multi-target assays into a single test.^1^ However, test dynamics can vary by target despite the overall favorable diagnostic performance and convenience of multiplex assays. The indiscriminate use of these multiplex panels can lead to false-positive results when applied to circumstances with a low pretest probability of an infectious syndrome.^2–4^

Prior studies have demonstrated the limited utility of multiplex gastrointestinal (GI) panels for hospitalized patients in the later part of their hospital stay.^5–8^ However, these conclusions were primarily based on general inpatient populations. The performance characteristics of multiplex GI testing among hospitalized immunocompromised patients remain less well characterized. Further, guidelines from the Infectious Diseases Society of America (IDSA) recommend multiplex testing for infectious gastroenteritis in immunocompromised hosts but provide no perspective on stewardship in this population.^9^ Therefore, validation of a safe and effective diagnostic stewardship approach for immunocompromised patients is essential in this high-risk patient population.

In the present study, we retrospectively evaluated the yield of the FilmArray GI panel among all pediatric and adult patients at a tertiary cancer center to describe the epidemiology of infectious diarrhea in an immunocompromised cohort in the hospitalized and ambulatory settings and to generate evidence that may inform diagnostic stewardship strategies among hospitalized oncologic patients.

## Methods

### Study population

Memorial Sloan Kettering Cancer Center (MSKCC) is a 514-bed tertiary cancer center in New York City with approximately 25,000 admissions and 173,000 annual patient-days. From February 2020 to January 2025, all FilmArray GI panels performed on adult and pediatric patients were included in the study. During this period, there were no institutional restrictions or clinical decision support tools in place to influence the ordering of the FilmArray GI panel and no rejection protocol for formed stool. *Clostridioides difficile* test results from the panel are not reported as the study institution utilizes an alternative testing pathway for *C. difficile*. Also, *Escherichia coli* O157 was only reported if Shiga-toxin-producing *E. coli* (STEC) was detected. Identification of case patients and their medical records at the time of stool sample collection were extracted from the electronic medical record.

### Study method

The primary outcomes analyzed were pathogen-specific positivity rate examined by host factors (absolute neutrophil count and prior bone marrow transplant) and test setting (community- vs hospital-acquired). For the latter, tests performed in the outpatient setting or hospital days 0–2 were categorized as community-acquired, and tests performed on hospital day 3 or later as hospital-acquired. At the study institution, stool samples are collected in a sterile container then transported to the clinical microbiology laboratory where the stool is transferred into Clary-Blair media. PCR test is then performed according to the manufacturer’s instructions.^10^

Secondary outcomes included clinically significant infection for five high-risk target pathogens (Cyclospora, Cryptosporidium, Salmonella, Giardia, and STEC) detected at or beyond hospital day 3 among neutropenic patients. Any patient with an absolute neutrophil count ≤500 k/µL on or within 3 days of the stool collection date was categorized as neutropenic. Clinically significant infection was determined through independent review of the electronic medical record by two infectious diseases physicians. A detection was considered clinically significant when compatible GI symptoms were present and the adjudicating physicians determined that the detected organism was the most likely explanation for the clinical syndrome. Reviews were conducted independently, and discrepant assessments were adjudicated by a third infectious diseases physician. All reviewers were study authors. Unique infection events were assessed in a secondary analysis that excluded repeat detection of the same organism within six months of a prior positive result. This six-month interval was selected as a pragmatic analytic definition intended to reduce the influence of persistent organism detection or prolonged shedding.

### Statistical analysis

Baseline demographic and clinical characteristics for all study patients were reported as absolute frequency and percentage. Performance metrics of the FilmArray GI panel, including positivity rate and pathogen distribution, were evaluated across subgroups defined by neutropenia status, receipt of hematopoietic stem cell transplantation (HSCT) or chimeric antigen receptor (CAR) T-cell therapy within the preceding year, and hospital admission status at the time of testing.

The MSKCC institutional review board approved this retrospective study and granted a waiver of informed consent.

## Results

### Baseline characteristics of the study cohort

During the 5-year study period, 29,727 FilmArray GI panels sent from 15,864 patients were available for analysis. Half the stool samples were collected from females and 13,735 (46.2%) from patients who were ≥65 years of age, whereas 16,803 (56.5%) were collected in the inpatient setting. Among samples collected in the inpatient setting, 4,117 (24.5%) had received a laxative in the preceding 3 calendar days. Nearly a quarter of patients were neutropenic at the time of sample collection (22.3%) and/or had received a HSCT or CAR T-cell therapy within the prior year (23.6%). The underlying cancers were 3,709 (23.4%) with hematologic malignancy and 12,027 (75.8%) with solid tumors (Table 1).

**Table 1.** Demographics and baseline characteristics of study patients

Characteristic	n (%)
Age ≥65 yr	13,735 (46.2)
Female sex	15,138 (50.9)
Inpatient at time of testing	16,803 (56.5)
Cancer type	
Hematologic malignancy	3,709 (23.4)
Solid tumor	12,027 (75.8)
Other	128 (0.8)
Laxative exposure within previous 3 days (inpatient only)	4,117 (24.5)
Chemotherapy within previous 30 days	10,257 (34.5)
Neutropenic ≤500 cells/µL within 3 days	6,636 (22.3)
BMT or CAR-T within previous year	7,015 (23.6)

Abbreviations: BMT, bone marrow transplant; CAR-T, chimeric antigen receptor T-cell.

### FilmArray GI panel positivity rate and target detection by location and population

**Figure 1.**
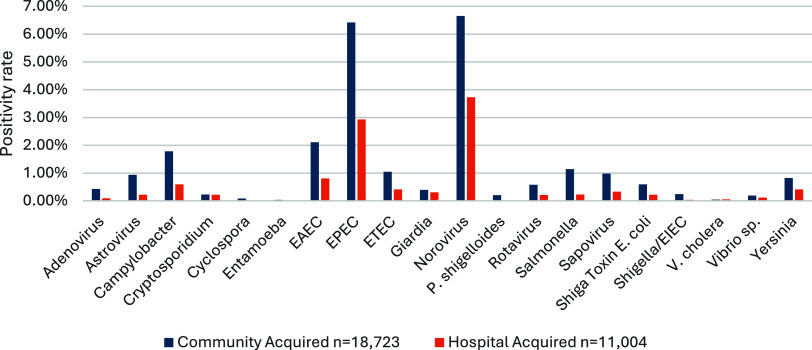
Detection rate by FilmArray GI panel targets among stools samples collected in the community-acquired versus hospital-acquired settings. EAEC, Enteroaggregative Escherichia coli; EIEC, Enteroinvasive Escherichia coli; EPEC, Enteropathogenic Escherichia coli; ETEC, Enterotoxigenic Escherichia coli; sp, species.

**Figure 2.**
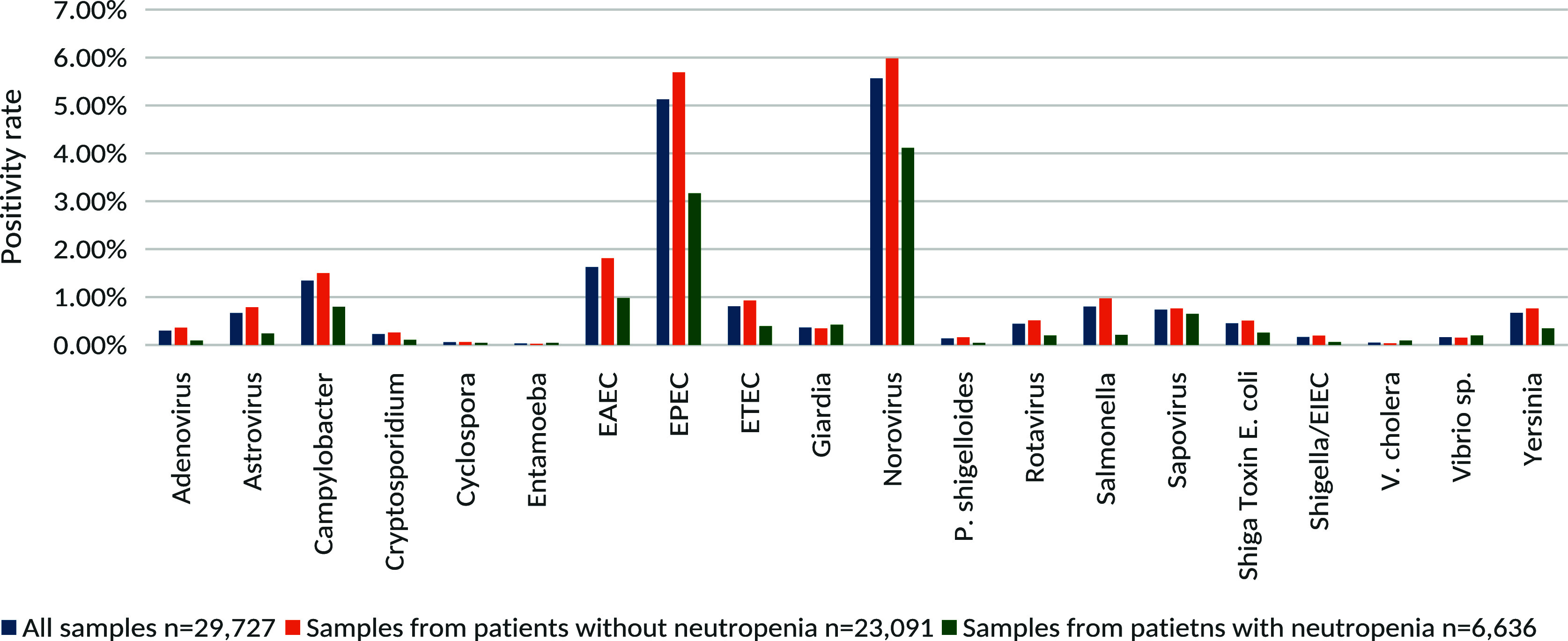
Detection rate by FilmArray GI panel targets among stools samples collected from patients with versus without neutropenia (ANC ≤500 k/µL). ANC, absolute neutrophil count; EAEC, Enteroaggregative Escherichia coli; EIEC, Enteroinvasive Escherichia coli; EPEC, Enteropathogenic Escherichia coli; ETEC, Enterotoxigenic Escherichia coli; sp, species.

**Figure 3.**
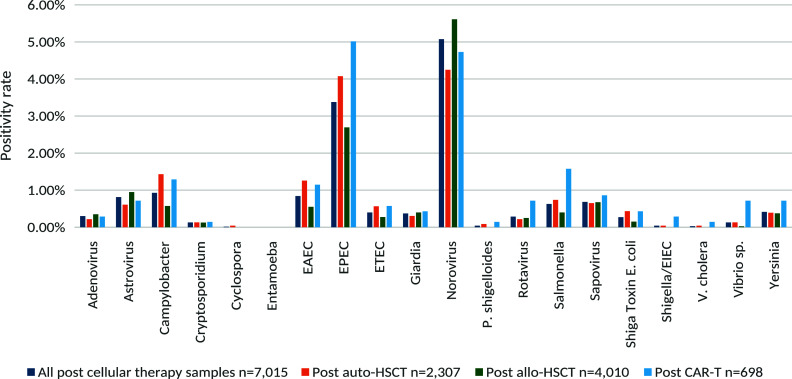
Detection rate by FilmArray GI panel targets among stools samples collected from patients post-cellular therapy. allo, allogeneic; HSCT, hematopoietic stem cell transplantation; CAR-T, chimeric antigen receptor T-cell; EAEC, Enteroaggregative Escherichia coli; EIEC, Enteroinvasive Escherichia coli; EPEC, Enteropathogenic Escherichia coli; ETEC, Enterotoxigenic Escherichia coli; sp, species.

The FilmArray GI panel yielded the greatest target detection rate in the outpatient setting at 23.4%, whereas the positivity rate during hospital days 0–2 and hospital days ≥3 were 15.6%, and 10.1%, respectively (Table 2). Across all tests analyzed, the most frequently detected targets were norovirus and Enteropathogenic *E. coli* (EPEC) in both the community and hospital settings with all remaining targets detected at rates below 2% (Figure 1). Similar pathogen distributions and positivity rate trends were observed among patients with neutropenia and among recipients of HSCT or CAR T-cell therapy, with the highest positivity rates occurring in the outpatient setting and progressively lower positivity rates later during hospitalization (Figures 2 and 3, Table 2).

**Table 2.** Positivity rate of FilmArray GI panel tests by patient location, neutropenia status, and recent cellular therapy. (A) represents all tests performed, (B) excludes positive shedding events from the numerator, and (C) excludes isolated EPEC and/or Norovirus detections from the numerator

A. All tests			
	Outpatient	Hospital Days 0-2	Hospital Days ≥3
**Positivity rate overall**	23.4%	15.6%	10.1%
**Positivity rate during episode of neutropenia**	19.6%	15.1%	8.7%
**Positivity rate within 1 year of cellular therapy**	23.7%	14.2%	8.2%

B. All tests excluding shedding events^a^			
	Outpatient	Hospital Days 0-2	Hospital Days ≥3
**Positivity rate overall**	20.6%	13.5%	8.1%
**Positivity rate during episode of neutropenia**	16.6%	12.8%	7.1%
**Positivity rate within 1 year of cellular therapy**	18.0%	9.6%	6.1%

C. All tests excluding isolated EPEC and/or Norovirus positivity			
	Outpatient	Hospital Days 0-2	Hospital Days ≥3
**Positivity rate overall**	11.9%	7.3%	4.1%
**Positivity rate during episode of neutropenia**	9.3%	7.1%	3.4%
**Positivity rate within 1 year of cellular therapy**	11.4%	7.2%	3.3%

a There were a total of 735 stool samples where the detection of at least one organism represented a shedding event. These were distributed evenly between the inpatient and outpatient samples.

### Clinical review of high-risk organisms among patients with neutropenia detected at or beyond hospital day 3

**Figure 4.**
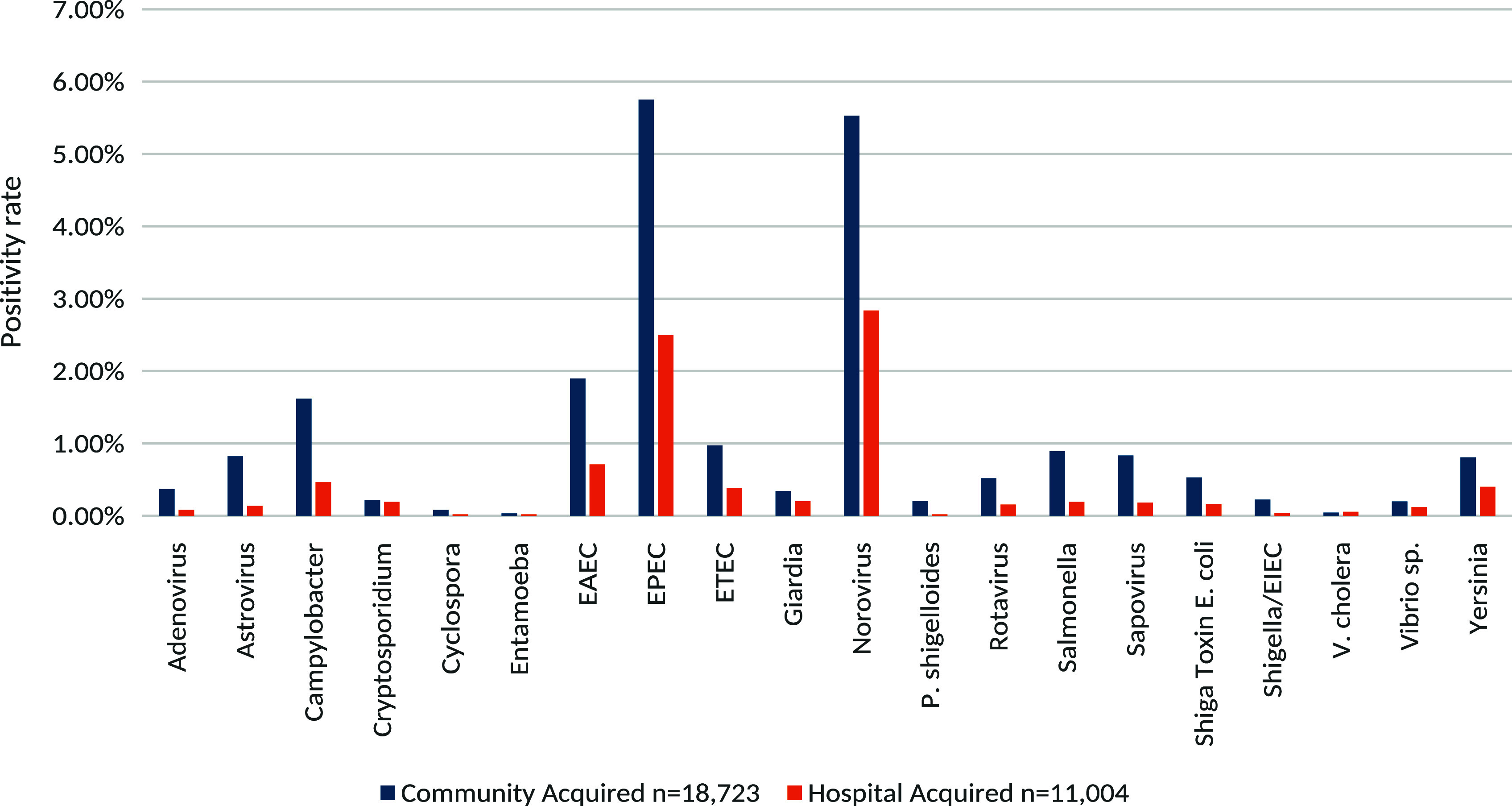
Detection rate by FilmArray GI panel targets among stools samples collected in the community-acquired versus hospital-acquired settings where pathogen shedding (defined as repeat positive within 6 months) was excluded from the count. EAEC, Enteroaggregative Escherichia coli; EIEC, Enteroinvasive Escherichia coli; EPEC, Enteropathogenic Escherichia coli; ETEC, Enterotoxigenic Escherichia coli; sp, species.

Among the 4,709 stool samples sent during an episode of neutropenia in the hospital-acquired setting, there were a total of 31 newly positive high-risk organisms detected. The breakdown was as follows: Cyclospora n = 0, Cryptosporidium n = 3, Salmonella n = 6, Giardia n = 16, and STEC n = 6. Blinded review of the electronic medical record by three infectious disease physicians found that three of the targets were clinically significant. All were Giardia, and none led to hemodynamic instability or escalation of care to the intensive care unit. When shedding events were excluded from the target count, the positivity rate for all organisms in the hospital-acquired setting decreased with the greatest absolute declines observed for EPEC and Norovirus at −0.43% and −0.89%, respectively (Figure 4).

## Discussion

In our study assessing the diagnostic utility of the BioFire FilmArray GI panel among nearly thirty thousand samples collected from immunocompromised cancer patients, the target detection rate was less than 2% for all pathogens other than norovirus and EPEC. There were no differences in the test yield among those with neutropenia or following cellular therapies compared to the overall cohort. Critically, the diagnostic yield was greatest in the community-acquired setting (21.0%) compared to the hospital-acquired setting (10.1%), and the decline in positivity rate from outpatient to hospital days 0–2 to hospital days ≥3 was preserved when analyzed by neutropenia status and recent cellular therapy.

IDSA guidelines recommend restricting enteric pathogen testing other than for *C. difficile* among patients who have been hospitalized >3 days due to low clinical yield.^9^ However, neutropenic and other immunosuppressed patient populations are frequently excluded from stewardship efforts due to concerns that these populations are at an increased risk of gastrointestinal infections and higher associated morbidity.^5,7^ Our study demonstrates that the low yield of the FilmArray GI panel was preserved in an immunocompromised patient population which included a substantial portion of neutropenic patients, HSCT or CAR T-cell, and recent chemotherapy recipients. Further, we show that the detection rate for all targets other than norovirus and EPEC was below 1%–2% regardless of underlying immune status. Regarding these two targets, it is important to note that most EPEC detections are of unclear clinical significance in cancer patients and transplant recipients and often lead to unnecessary antibiotic exposure.^11^ Similarly, the non-specificity of the norovirus target detection on multiplex assay is well established with up to a 27% false positivity rate.^12^ Overdiagnosis of norovirus gastroenteritis in a population with a high frequency of diarrhea leads to excessive use of treatments such as nitazoxanide.^13^ Although timely diagnosis of hospital-acquired norovirus cases is critical, the prevailing evidence suggests that single target tests are most optimal for reliable norovirus surveillance and diagnosis.^14,15^ Collectively, these findings provide evidence supporting consideration of a stewardship approach for the FilmArray GI panel among hospitalized patients ≥3 days without exclusion of immunocompromised hosts. Future studies should evaluate the implementation, safety, and effectiveness of such stewardship interventions in clinical practice.

Diagnostic stewardship of gastrointestinal multiplex PCR assays offers an important opportunity for antimicrobial conservation in an oncologic setting. The ability of multiplex panels to detect pathogen DNA from one or more targets and the fact that this does not necessarily indicate viable organisms can complicate antibiotic decision-making.^16–18^ We attempt to control for shedding of non-viable organisms with a secondary analysis that excludes repeat detection of the same pathogen. Further, qualitative studies and clinical impact analyses have demonstrated that unrestricted multiplex stool testing can drive inappropriate antibiotic use and increase financial burdens on the healthcare system without improving patient management, whereas targeted use can help streamline antibiotic use.^19–22^ Nearly one-quarter of inpatient stool samples in our study were obtained from patients who had received a laxative within the preceding three days. Future investigations should evaluate whether incorporation of recent laxative exposure into diagnostic stewardship algorithms could further improve test utilization and diagnostic yield among hospitalized oncology patients.

The are several limitations to our study. First, a positive FilmArray GI panel result was used as a proxy for clinically significant infection. However, we assess the performance of the FilmArray GI panel for the detection of clinically significant infection among hospitalized neutropenic patients who were newly positive for high-risk pathogens. Second, we report positivity rates based on tests and not episodes or unique patients. As chronic subclinical shedding of enteric pathogens is a recognized feature in immunosuppressed hosts, it is likely that many of the positive results represented in this study reflect chronic shedding and we may have therefore overestimated the diagnostic utility.^23–25^ Although we performed a secondary analysis excluding repeat detections of the same organism within six months, this interval was selected as a pragmatic analytic definition and is not a validated threshold to distinguish persistent shedding from reinfection. Duration of organism detection after gastrointestinal infection varies by pathogen and may be prolonged in immunocompromised hosts. As a result, some repeat detections within six months may have represented true reinfection, while some detections occurring beyond six months may have reflected persistent shedding. This analysis should therefore be interpreted as a sensitivity analysis intended to reduce the influence of repeated positive tests rather than as a definitive classification of colonization, shedding, or reinfection. Finally, the absence of an immunocompetent patient comparator arm limits more definitive statements regarding the feasibility of a stewardship approach to the FilmArray GI panel in the immunosuppressed oncology space. The performance of multiplex PCR assays for infectious gastroenteritis is variable across assays, hosts, and institutions, and independent validations are critical prior to local implementation of a stewardship initiative.

In summary, the BioFire FilmArray GI panel has low utility in the inpatient setting for severely immunosuppressed hosts. Our findings support the extension of commonly applied stewardship approach of limiting testing in immunocompromised patients who have been hospitalized for ≥3 days.
